# Four Functionally Distinct Regions in the Left Supramarginal Gyrus Support Word Processing

**DOI:** 10.1093/cercor/bhw251

**Published:** 2016-10-17

**Authors:** M. Oberhuber, T. M. H. Hope, M. L. Seghier, O. Parker Jones, S. Prejawa, D. W. Green, C. J Price

**Affiliations:** 1 Wellcome Trust Centre for Neuroimaging, Institute of Neurology, University College London, London, UK; 2 Cognitive Neuroimaging Unit, Emirates College for Advanced Education (ECAE), P.O. Box 126662, Abu Dhabi, UAE; 3 FMRIB (Oxford Centre for Functional MRI of the Brain), University of Oxford, Oxford, UK; 4 Wolfson College, University of Oxford, Oxford, UK; 5 Collaborative Research Centre 1052 “Obesity Mechanisms”, Faculty of Medicine, University Leipzig, Leipzig, Germany; 6 Department of Neurology, Max Planck Institute for Human Cognitive and Brain Sciences, Leipzig, Germany; 7 Experimental Psychology, Faculty of Brain Sciences, University College London, London, UK

**Keywords:** functional MRI, language, parietal lobe, phonological processing, speech production

## Abstract

We used fMRI in 85 healthy participants to investigate whether different parts of the left supramarginal gyrus (SMG) are involved in processing phonological inputs and outputs. The experiment involved 2 tasks (speech production (SP) and one-back (OB) matching) on 8 different types of stimuli that systematically varied the demands on sensory processing (visual vs. auditory), sublexical phonological input (words and pseudowords vs. nonverbal stimuli), and semantic content (words and objects vs. pseudowords and meaningless baseline stimuli). In ventral SMG, we found an anterior subregion associated with articulatory sequencing (for SP > OB matching) and a posterior subregion associated with auditory short-term memory (for all auditory > visual stimuli and written words and pseudowords > objects). In dorsal SMG, a posterior subregion was most highly activated by words, indicating a role in the integration of sublexical and lexical cues. In anterior dorsal SMG, activation was higher for both pseudoword reading and object naming compared with word reading, which is more consistent with executive demands than phonological processing. The dissociation of these four “functionally-distinct” regions, all within left SMG, has implications for differentiating between different types of phonological processing, understanding the functional anatomy of language and predicting the effect of brain damage.

## Introduction

Phonological processing allows us to detect, discriminate, represent, manipulate, and produce speech sounds. It therefore underpins multiple functions that are fundamental to speech comprehension, speech production (SP), and reading. Prior fMRI and PET studies have reported increased activation in the left supramarginal gyrus (SMG) when neurologically healthy participants make phonological decisions on visually presented words compared with semantic decisions on matched words. For example, when deciding whether a written word (e.g., “donkey” or “banana”) has 2 syllables or not (phonological decision), compared with deciding whether a written word refers to an animal or not (semantic decision). Activation in left SMG has also been reported for reading aloud pseudowords compared with reading aloud words. Moreover, there is a striking correspondence in the location of activation that is higher for (1) pseudoword than word reading and (2) phonological more than semantic decisions, with the average peaks across both types of studies located in an anterior dorsal part of left SMG (see Table 1 for review). Together these studies suggest that the anterior dorsal part of left SMG is involved in sublexical phonological processing of orthographic stimuli because this region is activated by both phonological decisions relative to semantic decisions on written words and by reading aloud written pseudowords relative to familiar words.

**Table 1:** SMG activation reported in prior studies of phonological decisions and pseudoword reading

Study	Technique	*x y z*	Mean* x y z*
*Reading aloud visual pseudowords > words*			
Vigneau et al. (2005)	fMRI	−60 −28 36	
		−52 −36 44	
Binder et al. (2005)	fMRI	−37 −37 37*	−49 −35 39
		−47 −38 41*	
Taylor et al. (2014)^a^	fMRI	−46 −38 44	
Carreiras et al. (2007)	fMRI	nr	
Cummine et al. (2013)	fMRI	nr	
Fiez et al. (1999)	PET	nr	
Herbster et al. (1997)	PET	nr	
Mechelli et al. (2000)	fMRI	nr	
Rumsey et al. (1997)	PET	nr	
*Phonological > semantic decisions on visual words*			
Scott et al. (2003)	PET	−60 −26 39*	
Mummery et al. (1998)	PET	−59 −31 38*	
Seghier et al. (2004)	fMRI	−55 −35 40*	−52 −35 40
Devlin et al. (2003)	fMRI	−42 −40 46	
Price et al. (1997)	PET	−42 −44 36*	
Roskies et al. (2001)	PET	nr	
*Phonological > perceptual decisions on visual words versus letter strings*			
Xu et al. (2002)	fMRI	−47 −44 33*	−43 −45 37
Seghier et al. (2004)	fMRI	−39 −46 42*	
Gitelman et al. (2005)	fMRI	nr	

^a^Excluded from mean coordinates (because not cluster peak).

The *x, y, z* coordinates of SMG activation reported in previous fMRI or PET studies of phonological decisions or pseudoword reading that used alphabetic stimuli. No SMG activation was observed when the stimuli were presented in the auditory modality (Shuster 2009) or when reading aloud pseudowords was compared with lexical decisions on pseudowords (Carreiras et al. 2007). When coordinates were given in Talairach space, they were converted to MNI space (*converted from Talairach space using the tal2icbm transformation, Lancaster et al. 2007). nr, no SMG coordinates reported.

The precise contribution of SMG activation in this process could arise at multiple different levels including: (1) the recoding of sublexical orthography-to-phonology; (2) phonological or auditory short-term memory to hold the sublexical phonological inputs in memory while they are integrated into a sequence; (3) executive processes (such as visual attention or the maintenance of task sets) that are not specific to phonological tasks but increase for more demanding tasks including phonological relative to semantic decisions (Mummery et al. 1998) and pseudoword relative to word reading (Binder et al. 2005); and (4) articulatory sequencing which may be more demanding for the unfamiliar phonological structure of pseudowords.

The current study examined evidence for each of the above alternatives. Furthermore, we investigated the possibility that different parts of the left SMG contribute to word processing in different ways. From prior literature, we note that increasing the demands on auditory short-term memory increases SMG activation at [−44, −38, 21] and [−63, −34, 19] (Buchsbaum and D'Esposito 2009; Koelsch et al. 2009) which is more ventral than the activation associated with phonological decisions and pseudoword reading (see Table 1). In contrast, executive processing has been associated with more posterior SMG activation at [−42, −47, 38] and [−45, −39, 42] (Ravizza et al. 2004; Hope et al. 2014). It is therefore possible that executive processing might explain why this posterior part of SMG has been reported for phonological decisions when the stimuli were words and the baseline was perceptual decisions on letter strings (see Table 1) which does not control for semantic, orthographic, or executive processing. In contrast, the more anterior dorsal SMG area is associated with phonological decisions after controlling for semantic, orthographic, and executive processing (see Table 1). This functional segregation, apparent in the neuroimaging literature, is supported by reports of a strong heterogeneity in connectivity patterns (Mars et al. 2011), cytoarchitectonic characteristics (Caspers et al. 2006), and receptor distribution within SMG (Caspers et al. 2013).

To investigate how SMG contributes to phonological tasks and whether there is within-subject evidence for the apparent functional dissociation along the anterior-posterior and dorsal-ventral directions in SMG, we compared fMRI activation over 8 overt SP tasks: reading and repeating familiar words (W), reading and repeating unfamiliar pseudowords (P), naming objects (O) from pictures or their sounds, naming colors of meaningless visual stimuli (visual baseline), or naming the gender of meaningless humming (auditory baseline). This experimental design allowed us to dissociate multiple different functions by independently manipulating the presence of sublexical phonological cues (words and pseudowords relative to objects and baselines); semantic content (words and objects relative to pseudowords and baselines), and stimulus modality (visual vs. auditory). In addition, all 8 conditions were repeated during a one-back (OB) matching task to test whether the observed effects in SMG were commonly or differentially involved in articulatory processes (during the SP conditions) and/or silent matching (during the OB matching task). Our rationale for testing our hypotheses is summarized below and in Table 2.

**Table 2:** Dissociating activation related to different types of processing

Main contrasts	Orthographic-to-phonological recoding	Phonological STM	Auditory STM	Executive processing	Articulatory sequencing	Lexical&sublexical integration
[P>WOB]	✓					
[WP>OB]		✓	✓			
[PO>WB]				✓		
[O>B]					✓	
[W>POB]						✓
*Masks*						
[P>W]	✓			✓		
[P>O]	✓	✓	✓			
[P>B]	✓			✓		
[P>R]	✓					
[W>P]						✓
[W>O]	✓	✓	✓		✓^a^	✓
[W>B]	✓					✓
[W>R]						✓
[O>W]				✓	✓^a^	
[O>B]				✓		
Modality effect	Vis	Vis&Aud	Vis (& Main Aud>Vis)	Vis&Aud	Vis&Aud	Vis&Aud

^a^Exclusive masks.

STM, short-term memory; P, pseudowords; W, words; O, objects; B, baselines; R, rest; Vis, visual; Aud, auditory; Main Aud>Vis, Main effect of auditory>visual stimuli.

### Recoding of Sublexical Orthography-to-phonology

If SMG activation reflected the demands on orthographic-to-phonological recoding, we would expect activation to be higher for (1) reading pseudowords than all other conditions and (2) reading words than pictures of objects. The pattern of activation across visual conditions was therefore expected to be P>W>O, irrespective of task (SP and OB matching). Moreover, this pattern of effects should be significantly greater in the visual modality than the auditory modality because orthographic processing is not explicitly required for any of the auditory tasks.

### Phonological or Auditory Short-term Memory

If SMG activation reflected the demands on phonological short-term memory, then we expect activation to be (1) higher for stimuli with phonological input (i.e., W&P>O&B) in both modalities and both tasks and (2) higher for pseudowords than words (P>W) because pseudowords are reliant on phonological processing whereas words are facilitated by lexical and semantic processing.

If SMG activation reflected the demands on auditory short-term memory, we would expect activation to be (1) higher for all auditory than all visual conditions in both tasks and (2) enhanced for visual stimuli that had the stronger auditory associations (i.e., the stronger phonological associations for words and pseudowords than objects and baselines, (Glaser and Glaser 1989).

### Executive Processing

If SMG activation reflected the demands on executive processing (e.g., attention), we would expect activation to increase for conditions that were more difficult. For example, reading pseudowords is more difficult than reading words because words but not pseudowords are facilitated by familiarity and semantic cues. Likewise, naming objects is more difficult than reading words because words but not objects are facilitated by sublexical phonological cues (Glaser and Glaser 1989; Binder et al. 2005). Behaviorally, difficulty is reflected by increased response times (RTs) and errors. Therefore, SMG activation that was related to difficulty (and executive processing) should mirror the effect on RTs and errors (P>W and O>W).

### Articulatory Sequencing

If SMG activation reflected the demands on articulatory sequencing, then we would expect SP activation to be (1) less for the baseline conditions which involved repetition of the same articulatory outputs (color names and genders) compared with all other conditions which involved constantly changing articulatory outputs; (2) the same for word and object naming conditions because articulatory output was controlled in these 2 conditions and (3) higher during SP than OB matching for all types of stimuli. The pattern of effects across conditions was therefore expected to be P&W&O>B and this effect was expected to be stronger during SP than the OB matching tasks that do not involve overt articulation.

## Materials and Methods

We report data from 2 fMRI paradigms (Paradigm 1 and Paradigm 2), which were conducted with 2 different groups of participants (Group 1 and Group 2), one for each paradigm. In Paradigm 1, there were 16 different conditions, 8 involving overt SP and 8 involving OB matching (see Table 3). This allowed us to look at stimulus by task interactions. Paradigm 2 included the same 8 SP conditions but not the 8 OB matching conditions. The data from Paradigm 2 contributed to the results in 2 ways: by replicating effects of interest during SP in Paradigm 1 using different subject cohorts and presentation parameters; and by providing responses times for the overt SP conditions which we were unable to extract from Paradigm 1.

**Table 3:** Experimental design

	Task	Stimulus modality	Response modality	Paradigm
(1)	Reading words (W)	Vis	SP	1 & 2
(2)	Reading pseudowords (P)	Vis	SP	1 & 2
(3)	Naming pictures of objects (O)	Vis	SP	1 & 2
(4)	Naming colors (B)	Vis	SP	1 & 2
(5)	Repeating words (W)	Aud	SP	1 & 2
(6)	Repeating pseudowords (P)	Aud	SP	1 & 2
(7)	Naming sounds of objects (O)	Aud	SP	1 & 2
(8)	Naming gender of voice humming (B)	Aud	SP	1 & 2
(9)	Word matching (W)	Vis	OB	1
(10)	Pseudoword matching (P)	Vis	OB	1
(11)	Object picture matching (O)	Vis	OB	1
(12)	Color matching (B)	Vis	OB	1
(13)	Word matching (W)	Aud	OB	1
(14)	Pseudoword matching (P)	Aud	OB	1
(15)	Sounds of objects matching (O)	Aud	OB	1
(16)	Gender matching (B)	Aud	OB	1

Vis, visual; Aud, auditory; SP, overt speech production; OB, one-back matching.

## Participants

A total of 85 participants were included in this study. Participant details for each group are provided in Table 4. All participants were native English speakers, right handed (assessed with the Edinburgh handedness inventory, Oldfield 1971) neurologically healthy and reported normal or corrected-to-normal vision and hearing. They gave written informed consent for participation and were compensated financially for their time. The study was approved by London Queen Square Research Ethics Committee.

**Table 4:** Experimental details

	Paradigm 1	Paradigm 2
***Participants***		
Number	26	59
Gender (n females/ n males)	12/14	34/25
Mean age in years (+/−SD)	31.44 (5.74)	44.5 (17.66)
***Stimulus properties***		
Stimulus duration in sec (+/−SD)	1.5	2.5
Visual stimuli	0.64 (0.10)	0.63 (0.09)
Auditory words^a^	0.68 (0.12)	0.65 (0.08)
Auditory pseudowords^a^	1.47 (0.12)	1.45 (0.15)
Sounds	1.04 (0.43)	1.05 (0.51)
Hums		
Average number of syllables (+/−SD)		
Reading words^a^	1.53 (0.68)	1.55 (0.68)
Repeating words^a^	1.53 (0.68)	1.68 (0.73)
Reading pseudowords	1.94 (0.92)	1.50 (0.51)
Repeating pseudowords^a^	1.90 (0.84)	1.50 (0.51)
Naming pictures^a^	1.55 (0.69)	1.48 (0.72)
Naming sounds	1.81 (0.92)	1.88 (0.94)
Naming gender	1.50 (0.51)	1.50 (0.51)
Naming colors	1.36 (0.49)	1.40 (0.50)
Average number of letters (+/−SD)		
Reading words^a^	5.24 (1.68)	5.08 (1.61)
Repeating words^a^	5.24 (1.68)	5.28 (1.38)
Reading pseudowords	5.28 (1.94)	4.40 (1.03)
Repeating pseudowords^a^	5.35 (1.72)	4.35 (1.08)
Naming pictures^a^	5.30 (1.75)	5.28 (1.75)
Naming sounds	5.64 (2.21)	5.65 (2.40)
Naming gender	5.00 (1.01)	5.00 (1.01)
Naming colors	4.89 (1.04)	4.80 (1.18)
***Timing parameters***		
ISI (sec)	2.52	2.5
Number of stimuli per block	9 (incl. one repeat)	10
Number of blocks per run	4	4
Total number of stimuli per run	36	40
Number of runs	16	8
Total time for each run (min)	3.2	3.4
Total acquisition time (min)	51.2	27.2
***Scanning parameters***		
TR (sec)	3.085	3.085
Number of slices	44	44
Number of volumes per run	62	66
Number of dummy acquisitions	5	5

^a^Across sets A, B, C.

## Previous Reports

Data from both paradigms were retrieved from the PLORAS Database (Seghier et al. 2016). All imaging and behavioral data from Paradigm 2 are novel and have not previously been reported. The Paradigm 1 data have previously been reported in studies of auditory word and pseudoword repetition (Hope et al. 2014; Parker Jones et al. 2014) and sublexical reading (Oberhuber et al. 2013). The figures and tables of results in Hope et al. (2014) reference dorsal SMG activation for task difficulty/executive processing effects (at Montreal Neurological Institute (MNI) [−45, −39, 42]) but do not report data from other parts of the SMG because they were not activated for auditory word repetition (the focus of that study). Likewise, Oberhuber et al. (2013) report the same dSMG [−42, −42, 45] area for both reading and repetition of pseudowords more than words but did not associate it with sublexical phonological processing because it was also more activated by object naming than word reading. Parker Jones et al. (2014) focus their analysis on a posterior ventral part of SMG known as TPJ or Spt [−51, −39, 21] and associate this region with auditory imagery independent of the presence or absence auditory input. Whole brain results, including those in other parts of SMG, were only reported for the comparison of pseudoword repetition with naming nonverbal sounds. Therefore, none of the data reported in these prior studies are able to answer the questions we focus on in the current study.

## Experimental Design

The 16 conditions in Paradigm 1 were organized in a 2×2×2×2 factorial design. Factor (I) “modality” manipulated the stimulus modality, i.e., auditory versus visual. Factor (II) was the presence or absence of semantic cues (words, pictures, and sounds of objects provide semantic cues, whereas pseudowords and meaningless baseline stimuli provide minimal or no semantic cues) and Factor (III) was the presence or absence of sublexical phonological cues (words and pseudowords contain sublexical cues, whereas pictures and sounds of objects and baseline stimuli do not, although they provide lexical phonological cues). Factor (IV) was response modality (SP vs. OB matching task). For the OB matching tasks, participants had to use their index/middle finger, on an MRI compatible button box, for a yes/no response to indicate if 2 consecutive stimuli are the same. In the auditory baseline, they were asked to attend to the gender of the voice and press one of 2 response keys.

Task difficulty was expected to be greater for pseudoword than word conditions (Binder et al. 2005) or for naming objects than words (Glaser and Glaser 1989). Therefore, task difficulty was least when both semantic and phonological information were present (i.e., for words).

Paradigm 2 manipulated Factors 1 to 3 but only included the 8 SP conditions, not the 8 OB matching conditions. In addition, all participants completed five other conditions that are not relevant to the current study. These were visual semantic decisions, auditory semantic decisions and production of sentences, verbs, and nouns.

## Stimulus Selection and Creation

Stimulus selection started by generating 128 pictures of easily recognizable animals and objects (e.g., “bus”, “elephant”, “plate”) with one to four syllable names. Written word stimuli were the written names of these 128 concepts. Auditory word stimuli were their spoken names recorded by a native English speaker with a southern British accent approximating Received Pronunciation. Pseudowords (e.g., golm) were created using a nonword generator (Duyck et al. 2004) and matched to the real words for bigram frequency, number of orthographic neighbors, and spoken word length. The nonverbal sounds associated with 32 of the objects (e.g., the sound of a telephone ringing or a dog barking) were taken from the NESSTI sound library (http://www.imaging.org.au/Nessti; Hocking et al. 2013). Object sound stimuli were not available, or not easily recognizable for the remaining 96 stimuli. The auditory baseline stimuli were recorded by male and female voices humming; therefore, removing any phonological or semantic content. Half the auditory baseline stimuli were matched in duration to the words (0.64 s) and the other half were matched in duration to the object sounds (1.47). It was not possible to match the object sounds and words because shorter sounds were not recognizable.

The visual baseline stimuli were meaningless object pictures, created by scrambling both global and local features, and then manually edited to accentuate one of 8 colors (brown, blue, orange, red, yellow, pink, purple, and green). Although the visual form and precise shade of the color stimuli changed on each trial, each of the 8 color names was repeated four times (32 stimuli in total). Consistent SP responses for each color and object were ensured for all stimuli in a pilot study conducted on 19 participants. See Table 4 for details on stimulus properties.

## Assigning Stimuli to Conditions and Counterbalancing in Paradigm 1

In Paradigm 1, each subject saw exactly the same stimuli in the SP and OB matching tasks. Half the subjects performed all 8 SP conditions first, the other half performed all the OB matching task first. Within each of these 2 groups, half were presented with auditory stimuli first, the other half were presented with visual stimuli first. Within each of these 4 groups, half responded to the OB matching task with fingers on their right hand and the other half responded with fingers on the left hand. Within these 8 groups, the order of the 4 types of stimuli (objects, words, pseudowords, and baselines) was presented in 4 different orders, making a total of 24 different subject orders. Post hoc analyses indicated that our condition dependent results were not significantly affected by whether the 8 SP conditions were performed before or after the OB matching conditions, although we did find a reduction of activation when stimuli had been seen before, which indicates an habituation effect.

The 128 object stimuli were divided into 4 sets of 32 (A, B, C, and D). Within each set, the 32 items were split into 4 blocks of 8 stimuli, with one of the 8 stimuli repeated in each block to make a total of 9 stimuli per block. In the OB matching tasks, the stimulus repeat needed to be detected and responded to. Set D included all the object concepts that had the most easily recognizable sounds (e.g., the sound of a telephone ringing). The remaining items were then assigned to sets A, B, or C attempting to control for as many stimulus variables as possible. Set D was always used for sound naming in the auditory modality and pseudoword reading in the visual modality. Sets A, B, and C were rotated across visual objects, reading words, and repetition of words. Therefore, these conditions were fully controlled for object names and concepts, and the demands on the motor execution of speech were matched. One of these sets was repeated for pseudoword repetition. Consequently, each set occurred an equal number of times, within subject and across the experiment.

The stimuli in Set D (i.e., those we had sounds for) had slightly more syllables on average (mean 1.8) than the other stimuli (mean 1.5). Post hoc tests confirmed that there was no significant effect of word length.

## Assigning Stimuli to Conditions and Counterbalancing in Paradigm 2

In Paradigm 2, all participants underwent the identical task order with no change in stimuli across participants. The motivation for this decision was to ensure that, when looking at inter-subject variability in future studies, task order and stimulus effects were held constant. For Paradigm 2, we selected those stimuli that produced the most consistent responses across participants in Paradigm 1. Importantly, none of the effects reported in the current paper can be attributed to order or stimulus effects because we looked for effects that were consistent across Paradigms 1 and 2. The order of the 13 tasks in Paradigm 2 was: (1) semantic decisions on pictures of objects, (2) naming 2 objects from pictures, (3) naming the action between 2 objects (e.g., eating), (4) sentence production from pictures, (5) semantic decisions on heard object names, (6) reading words, (7) repeating words, (8) naming objects from pictures, (9) naming colors, (10) naming sounds of objects, (11) reading pseudowords, (12) repeating pseudowords, (13) naming the gender of the voice humming. Each condition presented 4 blocks of 10 stimuli. Different sets of pseudowords were presented in the auditory and visual modality, with half the pseudowords in each set having 1 syllable and the other half having 2 syllables.

Object concepts were assigned to the 4 relevant conditions as follows: Those presented as written and auditory words had previously been presented as pictures in the first 5 tasks (Conditions 1 and 3 above); those presented as pictures had previously been presented as auditory words (Condition 5) or the sentence production task (Condition 2); and those presented as object sounds were a mix of those presented in other conditions. We also changed the visual baseline in Paradigm 2, reducing the number of colors to 5 (i.e., blue, orange, red, yellow, and green), to minimize errors that sometimes occurred when naming pink, purple, and brown. The names of these 5 colors were each repeated 8 times (40 trials in total). In the auditory baseline condition, male and female targets occurred 20 times each (40 trials in total). Within a condition, the effect of repetition (familiarity) on articulation was therefore highest for gender naming (20 repetitions of each response) and 8 times greater for color naming than the other conditions.

## Procedure

The procedures (i.e., out-of-scanner training, instructions, and stimulus presentation) were the same in Paradigm 1 and 2 but there were differences in timing parameters between the 2 paradigms that are listed in Table 4. Prior to scanning, each participant was trained on all tasks using a separate set of stimuli except for environmental sounds, which remained the same during training and experiment as this condition was more difficult and required more practice than the other conditions. The additional practice that participants had listening to the environmental sound stimuli could potentially influence activation (increased or decreased) related to practice or habituation. However, such effects would be specific to the auditory modality because participants were not familiarized with the object naming stimuli in the visual modality.

Participants were asked to produce an overt, single-word response for each stimulus while keeping their body and head as still as possible and their eyes open with fixation on the cross at the center of the screen. This was additionally monitored with eye-tracking during the auditory conditions.

Visual stimuli were each displayed for 1.5 s. The pictures subtended a visual angle of 7.4 degrees, with a screen resolution of 1024 × 768 (after scaling to 350×350 pixels). Words and pseudowords were presented in lower case Helvetica. Their visual angle ranged from 1.47 to 4.41 degrees with the majority of words (with 5 letters) extending 1.84 to 2.2 degrees.

Auditory stimuli were presented via MRI compatible headphones (MR Confon), which filtered ambient in-scanner noise. Volume levels were adjusted for each subject before scanning. Each subject's spoken responses were recorded via a noise-cancelling MRI microphone (FOMRI IIITM Optoacoustics), and transcribed manually for off-line analysis. The length of sound files varied across stimuli and tasks (see Table 4).

The script for stimulus presentation was written with COGENT (http://www.vislab.ucl.ac.uk/cogent.php) and run in MATLAB 2010a (MathWorks). Each of the tasks consisted of a separate run. Scanning started with the instructions “Get ready” written on the in-scanner screen, while 5 dummy scans were collected. This was followed by 4 blocks of stimuli. Each block was preceded by a written instruction (e.g., “Repeat”), lasting for the length of one TR each (i.e., 3.08 s), and followed by 16 s of fixation. The total length of each run was 3.2 min. An overview of the timing parameters is shown in Table 4.

The data acquisition per subject lasted an average of 1 h 30 min including out-of-scanner training, setting up, getting the subject into the scanner, structural, and functional imaging.

## fMRI Data Acquisition

Functional and anatomical data were collected on two 3 T scanners (both Trio, made by Siemens) using a 12 channel head coil. All subjects who completed Paradigm 1 were scanned on scanner A, whereas 30 subjects of Paradigm 2 were scanned on scanner A and 29 subjects on scanner B. Assignment was based on scanner availability. To minimize movement during acquisition, a careful head fixation procedure was used when positioning each participant's head in the 12 channel head coil. This ensured that none of the speech sessions were excluded after checking the realignment parameters. Functional images consisted of a gradient-echo EPI sequence and 3 × 3 mm in-plane resolution (TR/TE/flip angle = 3080 ms/30 ms/90°, EFOV = 192 mm, matrix size = 64 × 64, 44 slices, slice thickness = 2 mm, interslice gap = 1 mm). For Paradigm 1, we acquired 62 image volumes per time series, whereas for Paradigm 2 we acquired 66 volumes. For both Paradigms the number of volumes included 5 “dummy” scans to allow for T1 equilibration effects. The TR was chosen to maximize whole brain coverage (44 slices) and to ensure that slice acquisition onset was offset synchronized with stimulus onset, which allowed for distributed sampling of slice acquisition across the study (Veltman et al. 2002).

For anatomical reference, a high-resolution T1 weighted structural image was acquired after completing the tasks using a 3D modified driven equilibrium Fourier transform sequence (TR/TE/TI = 7.92/2.48/910 ms, flip angle = 16°, 176 slices, voxel size = 1 × 1 × 1 mm).

## Behavioral Data Processing for SP Tasks

Spoken responses were transcribed online and scored offline. For both Paradigms, each response was categorized as “correct” (i.e., when the response matched the target) or “incorrect” for all other trials (i.e., when the response did not match the target, was delayed or self-corrected).

Spoken responses were considered correct if they matched the target exactly or were almost identical in meaning (e.g., target = “mug”, response = “cup”). RTs for spoken responses in Paradigm 2 were obtained from the audio files. To compute them, we used an adaptive moving window filter that was tailored to each audio file. The optimal window length (i.e., the width which maximally smoothed the audio stream) was based on a portion of the respective audio file collected during rest. After smoothing the whole time series, we defined the onset of speech as a rise in the absolute amplitude of the smoothed audio stream beyond 1.5 standard deviation (SD) from the mean.

Behavioral data were analyzed in SPSS (IBM SPSS, NY, US). To test for main effects and interactions we conducted repeated measures 4×2 ANOVAs. Factor 1 was condition (words, pseudowords, objects, baseline stimuli) and Factor 2 was stimulus modality (visual vs. auditory). Greenhouse-Geisser correction was used because the assumption of sphericity was not met.

## fMRI Data Pre-processing

We performed fMRI data preprocessing and statistical analysis in SPM12 (Wellcome Trust Center for Neuroimaging, London, UK), running in MATLAB 2012a (Mathsworks, Sherbon, MA, USA). Functional Volumes were spatially realigned to the first EPI volume and unwarped to compensate for nonlinear distortions caused by head movement or magnetic field inhomogeneity. We used the unwarping procedure in preference to including the realignment parameters as linear regressors in the first-level analysis because unwarping accounts for nonlinear movement effects by modeling the interaction between movement and any inhomogeneity in the T2* signal. After realignment and unwarping, we checked the realignment parameters to ensure that participants moved less than one voxel (3 mm) within each scanning run. The anatomical T1 image was co-registered to the mean EPI image which had been generated during the alignment step and then spatially normalized to the MNI space using the new unified normalization-segmentation tool in SPM12. To spatially normalize all realigned EPI scans to MNI space, we applied the deformation field parameters that were obtained during the normalization of the anatomical T1 image. The original resolution of the different images was maintained during normalization (voxel size 1 mm^3^ for anatomical T1 and 3×3×3 mm^3^ for EPI images). After the normalization procedure, functional images were spatially smoothed with a 6 mm full-width half-maximum isotropic Gaussian kernel to compensate for residual anatomical variability and to permit application of Gaussian random-field theory for statistical inference (Friston et al. 1995). Each pre-processed functional volume was individually inspected for oddities before statistical analyses.

## First-level Analysis

Data from each task for each participant were entered into a subject specific, fixed-effect analysis using the general linear model (Friston et al. 1995). All stimulus onset times, for all conditions, were modeled as single events. For Paradigm 1, we used 2 regressors per task, one modeling instructions, and the other modeling each stimulus. For Paradigm 2, stimuli with correct responses were modelled separately from stimuli with incorrect or “other” responses (delayed, no response, or self-corrected).

Stimulus functions were convolved with a canonical hemodynamic response function. To exclude low-frequency confounds, the data were high-pass filtered using a set of discrete cosine basis functions with a cut-off period of 128 s. The contrasts of interest were generated for each of the 8 conditions relative to fixation.

## Effects of Interest

### Analysis 1: Activation During 8 SP Tasks Across 2 Paradigms

At the second level, we entered 16 contrasts, 8 for each Paradigm, into an ANOVA in SPM12, with Paradigm as a between subject factor and 8 conditions as a within subjects factor. Factorial main effects and interactions were entered at the second level contrast stage. Activation related to the effects of interest are identified below where P, pseudo-word; W, word; O, object naming; B, baseline; R, rest (see Table 2 for summary). Activation related to:

1. “Orthographic-to-phonological recoding” was identified by comparing pseudowords to all other visual stimuli (P>WOB) and inclusively masking this contrast with P>W, P>O, P>B, P>R, W>O, and W>B (see Table 2). We also searched for SMG activation that was higher for visual P&W than visual O&B and all auditory conditions.
2. “Phonological or auditory short-term memory” was identified by the main effect of sublexical phonological cues (i.e., W&P>O&B) inclusively masked by W>O and P>O. Activation related to auditory but not phonological short-term memory was expected to be greater for all auditory conditions than all visual conditions.
3. “Executive processing” was identified by comparing P&O>W&B and inclusively masking this contrast with P>W, O>W, P>B, and O>B.
4. “Articulatory sequencing” was identified by comparing object naming to baseline conditions (O>B) excluding activation that differed for O and W (that have matched articulatory output).

Each of these effects was repeated across modalities and in each modality separately. If an effect was only found in one modality, we tested for the modality by effect interaction.

In addition, our experimental design allowed us to test whether any parts of SMG were more activated for words than all other stimuli (W>P&O&B), inclusively masked with W>P, W>O, W>B, and W>R. Such effects cannot be attributed to semantic processing (which is expected to be higher for objects than words). Nor can it be attributed to sublexical phonological processing (which is expected to be higher for pseudowords than words). We therefore associated activation that was greatest for words with the integration of sublexical with lexical (or semantic) inputs.

## Statistical Thresholds

For the 5 effects of interest described above, we set the statistical threshold to *P* < 0.05 after family wise error (FWE) correction for multiple comparisons across the whole brain. The threshold for all masks (inclusive and exclusive) was consistently set at *P* < 0.05 (uncorrected).

### Analysis 2: Identifying the Effect of SP Within Regions of Interest from Analysis 1

This post hoc analysis was based on the subjects who performed both the SP and OB matching tasks (i.e., Paradigm 1). One of the 26 subjects was excluded due to a technical failure during OB matching on auditory words. Using data from the remaining 25 subjects, we entered 16 contrasts (8 contrasts for SP tasks and 8 contrasts for OB matching tasks), into a within-subjects one-way ANOVA. Using SMG regions of interest from Analysis 1, we tested how the effects identified in Analysis 1 (see above) interacted with task (SP > OB matching tasks).

## Results

### Behavioral Results

**Figure 1.**
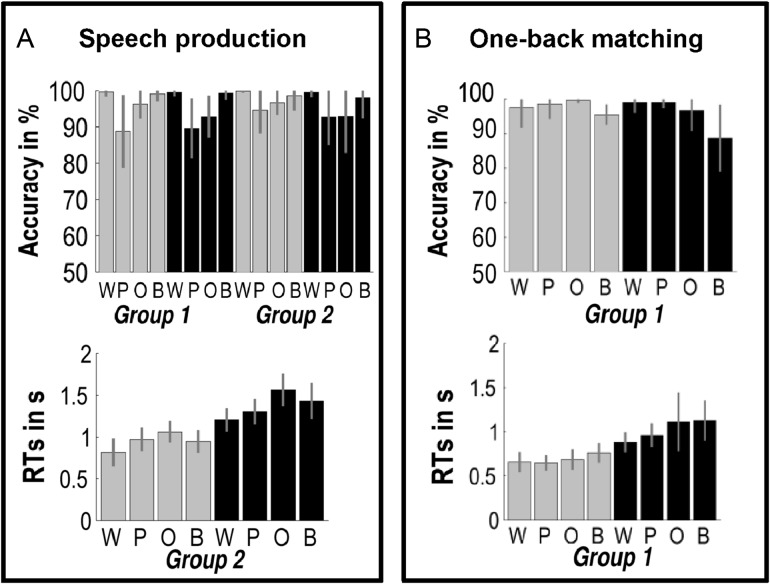
Behavioral data (mean with SD). See Table 3 for abbreviations; Gray/black = visual/auditory tasks. Note: RTs are for correct trials only and include stimulus delivery (longer for auditory than visual).

For SP tasks (see Fig. 1, Box A), in-scanner accuracy for both Paradigms was 98% or above for the word and baseline conditions; and 93% or above for object naming. Accuracy for pseudowords was higher for Paradigm 2 (94%) than Paradigm 1 (89%) because of changes to the stimuli (see Methods). Accuracy scores were computed after 3 outliers (subjects with less than 50% accuracy) had been removed (*n *= 2 from Paradigm 1 and *n* = 1 from Paradigm 2).

RTs for SP (Paradigm 2 only after 2 participants were excluded because their RTs were missing for one condition) were slower for auditory than visual stimuli because stimulus delivery was sequential for auditory stimuli but simultaneous for visual stimuli. Within modality, RTs were fastest for words and slowest for object naming. The effects of [O>W] and [O>P] are stronger in the auditory modality (F(1,56) = 15.15, *P* < 0.001 and F(1,56) = 33.51, *P* < 0.001, respectively). The effect of [P>W] is stronger in the visual modality (F(1,56) = 8.92, *P* = 0.004).

For OB matching (see Fig. 1, Box B, for details), behavioral scores for all 8 tasks are based on 22 subjects. The remaining 3 subjects had missing data from one of the OB matching conditions and were excluded from all behavioral analyses. As these subjects performed accurately in all the other conditions, we did not exclude these subjects from the fMRI analyses. Accuracy was above 98% for words, pseudowords, and objects, 96% for the visual baseline and 89% for the auditory baseline. In the RTs for correct trials only, we found a main effect of stimulus modality (as in SP), presumably because auditory stimuli were delivered sequentially rather than simultaneously (F(1,21) = 150.51, *P* < 0.001). In addition, we found that RTs are higher for the visual baseline compared with visual words (T(21) = 6.34, *P* < 0.001), pseudowords (T(21) = 5.49, *P* < 0.001), and objects (T(21) = 3.84, *P* < 0.001) and also for the auditory baseline compared with auditory words (T(21) = 6.89, *P* < 0.001) and pseudowords (T(21) = 4.93, *P* < 0.001), but not compared with objects (T(21) = 2.95, *P* = 0.777).

## fMRI Results

**Figure 2.**
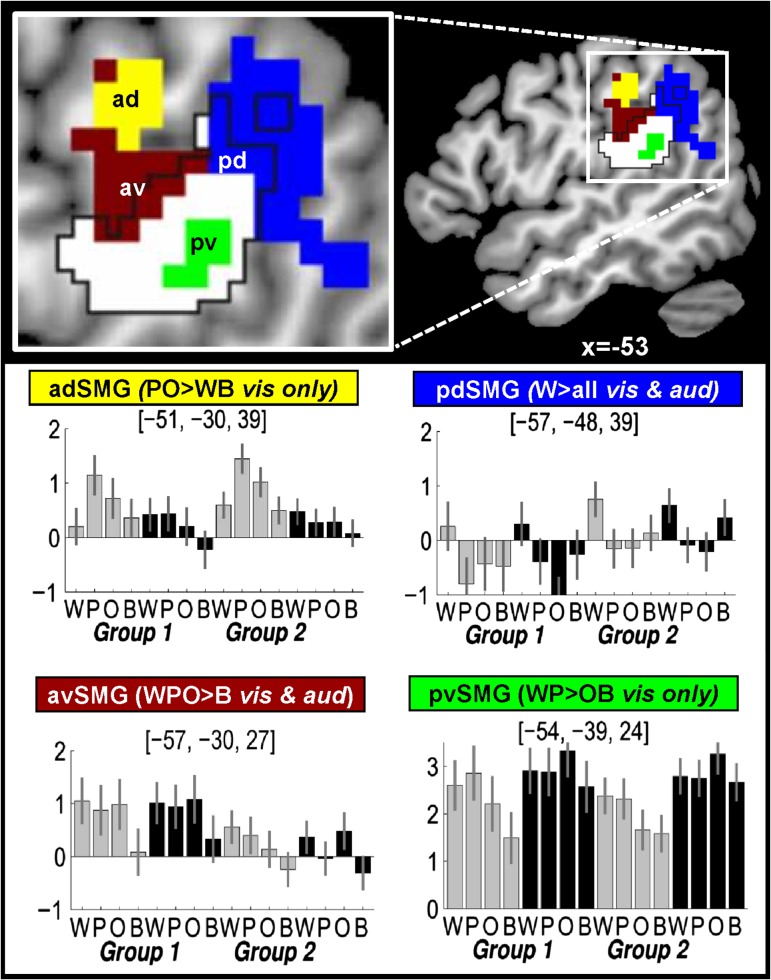
Functional subdivisions within the SMG. Top row shows the left hemisphere activation clusters (yellow, blue, brown, and green) within the SMG for each effect of interest (see plots for anatomical region and condition effects). The white area (outlined in black) shows the borders of the SMG according to the IBASPM software (http://www.thomaskoenig.ch/Lester/ibaspm.htm) in SPM 12 but other studies (see Table 1) include more anterior areas, as shown in yellow. The peak coordinates for each effect are reported in Table 3. The extent of activation includes voxels that were significant at *P* < 0.001 for the main effect of interest, and inclusive/exclusive masking at *P* < 0.05 uncorrected. Plots show the relative activation (with 90% confidence intervals) across all 8 conditions for Group 1 and Group 2. Gray/black bars = visual/auditory tasks. See Table 5 for abbreviations and other peak coordinates.

We focus on differential responses within SMG during SP (Analysis 1) and then report the task by condition interactions (Analysis 2). For Analysis 1, there were no significant group by condition interactions; therefore, we average over Paradigms for the statistics (Table 5) and illustrate the replication of effects in Fig. 2. Effects outside the left SMG are reported in Table 6.

**Table 5:** Location and significance of fMRI activation within left SMG for each type of processing during SP conditions

1) Auditory short-term memory (main effect of sublexical phonological input in the visual modality)												
	*k*	*x*	*y*	*z*	**[WP>OB]**	Int.	[P>O]	[P>B]	[W>O]	[W>B]	OB Aud>OB Vis	
pvSMG	189	−54	−39	24	5.2	4.2	3.3	5.3	3.1	5.0	*Inf*	
2) Executive processing (visual pseudowords & objects > words & baselines)												
	*k*	*x*	*y*	*z*	**[PO>WB]**	Int.	[P>W]	[P>B]	[O>W]	[O>B]		
adSMG	191	−51	−30	39	7.8	4.8	7.0	6.9	3.5	4.5		
		−39	−33	42	6.6	4.5	7.5	5.1	3.7	3.8		
3) Articulatory sequencing (all conditions > baselines)												
	*k*	*x*	*y*	*z*	**[O>B]**^a^	[W>B]	[P>B]					
avSMG	106	−54	−33	27	6.7	7.4	5.5					
4) Integrating lexical and sublexical phonological inputs (words > all other)												
	*k*	*x*	*y*	*z*	**[W>POB]**	[W>P]	[W>O]	[W>B]	[W>R]	[R>P]	[R>O]	[R>B]
pdSMG	250	−57	−48	39	7.7	7.1	7.4	5.2	5.5	4.1	4.1	n.s.
		−54	−51	42	7.7	6.9	7.5	4.4	4.3	3.5	4.2	n.s.

^a^Exclusively masked with [O>W] and [W>O] to exclude regions showing other effects of interest.

The columns show, from left to right, the location of the effect in left SMG (a, anterior; p, posterior; d, dorsal and v, ventral), k, cluster size; x y z, MNI coordinates. Z scores for statistical comparisons of different conditions (W, words; P, pseudowords; O, objects; B, baseline; R, rest) across auditory (Aud) and visual (Vis) modalities or for visual only (when stated). Int, Z score for the interaction of modality (i.e., visual/auditory) with the effect of interest. Inf, infinitive; n.s., not significant; L, left hemisphere. Z scores above 4.7 were significant at *P* < 0.05 following FWE correction for multiple comparisons across the whole brain. Those above 3.09 were significant at *P* < 0.001 uncorrected.

**Table 6:** Condition-specific SP effects outside left SMG

LH region	*x*	*y*	*z*	*k*	RH region	*x*	*y*	*z*	*k*
1) [WP>OB]									
Superior frontal gyrus	−9	57	21	9	Superior frontal gyrus	18	54	30	55
Precentral gyrus	−57	6	24	66	Temporal pole	45	12	−30	28
	−57	9	15		Middle temporal gyrus	48	−39	0	232
	−57	−21	27	57		63	−36	3	
Superior temporal sulcus	−54	−15	−3	11		60	−24	−3	
Putamen	−24	3	3	166	Putamen	24	3	6	101
	−33	0	9						
	−27	−21	6						
2) [PO>WB]									
Pars opercularis	−42	6	27	1350	Pars orbitalis	36	18	9	223
	−42	3	36			36	21	0	
*Insula*	−33	24	0			33	18	−9	
Middle temporal gyrus	−36	−72	12	7	Precentral gyrus	30	−6	48	17
Inferior temporal gyrus	−45	−66	−9	143		42	−3	42	
	−39	−42	−15		Superior parietal lobe	18	−60	51	77
	−45	−54	−24	7		24	−51	57	
Superior parietal lobe	−21	−63	51	96	*Postcentral Gyrus*	36	−36	48	
Occipital gyrus	−30	−90	−3	10	Supramarginal gyrus	42	−27	45	25
Thalamus	−9	−27	−9	42	Hippocampus	36	−15	−12	13
	−6	−18	−12		Amygdala	24	−3	−15	14
						18	−9	−9	
					Thalamus	0	−6	6	11
						12	−24	−12	18
					Cerebellum 6	27	−63	−27	480
						33	−54	−27	
						−3	−69	−27	
3) [O>B]									
Pars triangularis	−48	30	9	200	Frontal operculum	42	−21	27	29
	−42	33	3			42	−9	24	
Pars orbitalis	−36	30	−15		Anterior cingulate	0	39	−9	77
Superior frontal gyrus	−6	36	18	74		9	45	−3	
	−6	39	30		Middle cingulate	6	12	30	16
*Anterior Cingulate*	−6	12	36		Insula	45	0	12	39
Postcentral gyrus	−30	−36	69	59	*Superior Temporal*	51	3	6	
	−18	−39	75		*Gyrus*	36	9	12	
Middle cingulate	0	3	42	56	Cerebellum 6	15	−63	−21	98
	−3	−9	39			21	−57	−21	
	−6	−36	42	516	*Vermis*	6	−42	−18	
	−15	−27	39						
	18	−42	51						
Posterior cingulate	−6	−57	18	81					
	−6	−48	15						
	9	−51	18						
Angular gyrus	−33	−69	39	46					
	−33	−81	42						
	−39	−75	33						
Middle temporal gyrus	−51	−15	−9	29					
	−51	−6	−15						
Insula	−33	9	9	46					
	−39	3	12						
Putamen	−30	−15	−6	32					
	−39	−3	−6						
Thalamus	−15	−12	18	26					
Cerebellum 4/5/6	−15	−63	−18	59					
	−12	−51	−15						
	−18	−42	−24						
4) [W>POB]									
Superior frontal gyrus	−6	54	18	269	Superior frontal gyrus	3	51	30	269
	−6	45	33		Precentral gyrus	57	−3	27	78
Middle frontal sulcus	−36	15	48	100	Supplementary motor area	0	−15	57	535
	−30	21	39		*Middle Cingulate*	6	−30	54	
Precentral gyrus	−54	−6	30	113		−6	−27	45	
	−54	−6	18		Putamen	27	−9	6	38
Postcentral gyrus	−18	−30	63	85					
Posterior cingulate	−3	−51	24	86					
	9	−45	27						
	−12	−51	27						
Middle temporal gyrus	−48	−24	−12	99					
	−60	−18	−9						
	−60	−51	−3						
Putamen	−27	−6	3	120					
	−27	−18	9						
	−36	−18	18						

See Tables 3 and 5 for abbreviations. RH, right hemisphere; LH, left hemisphere. Significance level for masks: *P *= 0.05 (uncorrected). All effects are significant at *P* < 0.05 corrected for multiple comparisons, peak and/or extent using the FWE correction.

### Recoding of Sublexical Orthography-to-phonology

We did not find any SMG region where the pattern of activation across conditions corresponded to that expected for processing related to the translation of orthography into phonology (i.e., P>W>O&B in the visual > auditory modalities). Nor did we find SMG activation that was higher for visual P&W than visual O&B and the auditory conditions.

### Phonological or Auditory Short-term Memory

**Figure 3.**
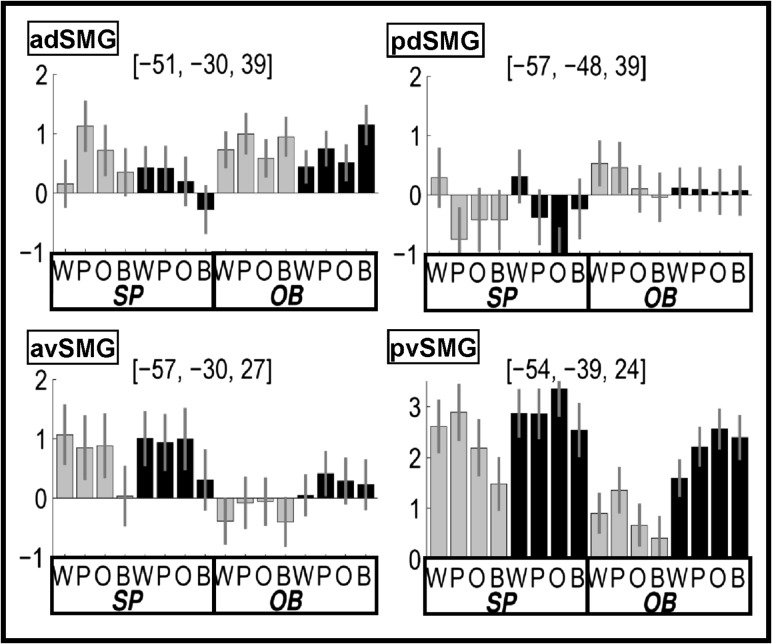
Task by condition effects in regions of interest. Plots show the relative activation (with 90% confidence intervals) during 8 SP and 8 OB matching tasks, at coordinates identified for condition effects during speech production tasks (Analysis 1). Gray/black bars = visual/auditory tasks. See Table 5 for abbreviations and text for significant interactions between task and condition.

Stimuli with sublexical phonological input (i.e., W&P>O&B) enhanced activation in the posterior ventral SMG (pvSMG) but only in the visual modality. This modality specific effect was confirmed by a significant interaction between [W&P>O&B] and stimulus modality. The OB matching tasks (Analysis 2, Fig. 3) replicated the effect of sublexical phonological input (W&P>O&B) in pvSMG in the visual modality. The response in this region was more consistent with auditory short-term memory than phonological short-term memory because (1) there was a main effect of all auditory versus all visual stimuli irrespective of phonological content (Z score = Inf); and (2) activation was not higher for pseudowords than words.

### Executive Processing

Reading pseudowords and naming objects compared with reading words and the visual baseline increased activation in an anterior dorsal SMG (adSMG) that extended posteriorly into the inferior parietal sulcus. This pattern of effects was only observed in the visual modality, and consequently, there was a highly significant interaction between P&O>W&B and stimulus modality (visual>auditory), see Table 5. Greater activation for pseudowords than words, with no significant difference between pseudoword reading and object naming was previously reported in Oberhuber et al. (2013).

In the OB matching task (Analysis 2, Fig. 3), activation in adSMG was higher for visual pseudowords than words (as observed for speech production) but not for objects than words. We also note that adSMG activation was highly significant for OB matching in both the visual baseline relative to rest (Z score = 5.3) and auditory baseline relative to rest (Z score = 6.4) and that these non-phonological effects were not significantly different (*P *> 0.01) from that observed during pseudoword reading relative to rest (Z score = 5.0), consistent with the longer RTs in these conditions.

### Articulatory Sequencing

We found that the greater demands on phonological output during W, P, and O compared with the baseline conditions increased activation in an anterior part of ventral SMG (avSMG) for both stimulus modalities.

During the OB matching tasks (Analysis 2, Fig. 3), there was no significant activation in avSMG for any condition. Consequently, of avSMG activation was significantly higher for speech production more than OB matching (Z score = 4.5) and this was qualified by an interaction between task and condition (W&P&O>B; Z score = 4.0).

### The Integration of Lexical and Sublexical Phonology

Activation in a lateral part of the posterior dorsal SMG (pdSMG) was more activated by words than all other stimuli, irrespective of whether the stimuli were presented in the visual and auditory modalities (see Table 5). This resulted in a 2-way interaction between sublexical phonological inputs and semantics (Z score = 4.7 at [−57, −48, 45]) because the effect of sublexical phonological inputs was greater (in pdSMG) in the context of semantics (W>O) than the absence of semantics (P>B).

In Analysis 2, we observed a task (SP > OB matching) by condition (W>P&O&B) interaction (Z score = 3.4). We also found a three-way interaction between phonological input, semantic content (W>P), and task (speech production > one back matching) (Z score = 3.5) at [−57, −48, 42].

In summary, we have distinguished the response in 4 different parts of SMG:

1. The pvSMG was activated for stimuli with phonological input (i.e., words and pseudowords) in the visual modality irrespective of task (speech production and OB matching). It was also strongly activated by auditory relative to visual stimuli during the OB matching task and for speech production relative to OB matching on the visual stimuli. This is consistent with our expected activation pattern for auditory short-term memory.
2. A region spreading from adSMG to inferior parietal sulcus was more activated for reading pseudowords and naming pictures than words. This is not consistent with a role in phonological input processing but rather with a role in executive processing.
3. A more avSMG was (1) associated with articulatory sequencing because it was more activated for words, pseudowords, and object naming relative to the baseline conditions in both modalities during the speech production tasks, and (2) not significantly activated during OB matching.
4. A lateral part of pdSMG was most activated for words (across modality) but only during the speech production tasks.

The region by condition interactions for this functional segregation are reported in Table 7.

**Table 7:** Region × condition analysis for speech production tasks

Regions		Conditions	Statistics	
adSMG vs	pdSMG	P – W	F(1,84) = 196.62	*P < 0.001*
	avSMG		F(1,84) = 112.83	*P < 0.001*
	pvSMG		F(1,84) = 93.570	*P < 0.001*
pdSMG vs	avSMG	W – P	F(1,84) = 37.95	*P < 0.001*
	pvSMG		F(1,84) = 15.51	*P < 0.001*
	pdSMG		F(1,84) = 196.62	*P < 0.001*
avSMG vs	pdSMG	O – B	F(1,84) = 10.47	*P < 0.002*
	pvSMG		F(1,84) = 13.69	*P < 0.001*
	adSMG		F(1,84) = 2.76	*P < 0.100*
pvSMG vs	pdSMG	P – O	F(1,84) = 31.25	*P < 0.001*
	avSMG		F(1,84) = 13.19	*P < 0.001*
	adSMG	W – B	F(1,84) = 37.73	*P < 0.001*
	pdSMG		F(1,84) = 5.18	*P < 0.025*

See Tables 3 and 5 for abbreviations.

Finally, the analysis of data from Paradigm 2 was repeated after including the mean response time per condition per subject as a covariate of interest. This did not affect the significance of phonologically driven SMG activation; and we found no evidence that SMG activation was affected by RTs either across or within conditions. Outside SMG, a main effect of response time across conditions was observed in bilateral insula, left middle temporal sulcus, cerebellum, and other areas.

## Discussion

Prior studies have highlighted the importance of the left SMG for phonological processing by comparing activation for either phonological to semantic decisions or nonword reading to word reading. The current study investigates the cause of left SMG activation during phonological tasks in more detail after controlling for multiple types of non-phonological processing (e.g., orthographic processing, articulatory sequencing, auditory short-term memory). We show that the anterior dorsal part of SMG, that has previously been associated with phonological processing (Table 1), was better explained by executive rather than phonological processes. In addition, we dissociate 3 other functionally distinct regions within left SMG that all contribute to word processing. An anterior ventral part of SMG responded to the demands on phonological output (articulatory sequencing) whereas a posterior ventral part of SMG was sensitive to phonological input and auditory processing of all types of stimuli, and a posterior dorsal part of SMG was most responsive to production of words that carry both lexical and sublexical phonological inputs. Below we discuss each of the 4 subregions in detail.

## Posterior ventral SMG

PvSMG was activated for the main effect of sublexical phonological input in the visual modality (i.e., more activation for written words and pseudowords than objects and baseline stimuli) irrespective of the mode of output (speech production or OB matching). In the auditory modality, this effect was reversed with more activation for auditory object sounds than any other condition. It cannot be explained in terms of (1) orthographic-to-phonological processing because activation was not higher for visual words and pseudowords than auditory words and pseudowords; (2) sequencing sublexical phonological codes because activation was not higher for articulating unfamiliar pseudowords than familiar words or (3) phonological short-term memory because activation was not higher for stimuli with phonological input (i.e., words and pseudowords) across tasks and modalities; and also not higher for pseudowords than words as expected given the greater demands on sublexical phonological cues.

Turning now to the prior literature, we note that the MNI coordinates of the pvSMG region that we found was more activated by visual words and pseudowords than visual objects or baselines [−54, −39, 24], correspond almost exactly to those we have previously associated with auditory imagery [−51, −39, 21] in Parker Jones et al. (2014), using the same data but a different set of contrasts (Paradigm 1, Analysis 2). In brief, in Parker Jones et al. (2014), we refer to pvSMG as TPJ (temporo-parietal junction) or Spt (posterior Sylvian fissure at the parietal–temporal boundary). Our conclusion was that this region is involved in the auditory representation of sounds (verbal or nonverbal) that can either be accessed bottom up via auditory inputs or top down in the absence of auditory inputs. Evidence of bottom up auditory processing is provided by the main effect of auditory versus visual OB matching (Z score = Inf). Evidence for top-down auditory processing comes from the main effect of phonology during silent visual OB matching (and prior studies of auditory imagery discussed in Parker Jones et al. 2014). The argument is that both bottom-up and top-down activation of auditory representations may contribute to pvSMG/TPJ/Spt activation during speech production.

On the basis of the conclusion that the pvSMG/TP/Spt region is involved in the auditory representation of sounds (Parker Jones et al. 2014), we suggest that enhanced pvSMG activation in the current study for sublexical phonological inputs in the visual modality is because written words and pseudowords have stronger auditory associations (from highly familiar sublexical phonological content) than pictures of objects or meaningless visual inputs. This interpretation is in line with other studies associating pvSMG activation with the demands on auditory memory for verbal and nonverbal material (Buchsbaum and D'Esposito 2009; Koelsch et al. 2009) but stands in contrast to the conclusions of Papoutsi et al. (2009) who interpreted increased ventral SMG activation at [−56, −38, 20] for repetition of pseudowords with 4 syllables compared with 2 syllables in terms of demands on syllabification and segmentation. We do not think that pvSMG activation in our study can be interpreted in terms syllabification and segmentation because this would result in higher pvSMG activation for pseudoword production than object naming, which we did not observe. On the other hand, the Papoutsi et al. (2009) findings can be re-interpreted in terms of the demands on auditory short-term memory because participants in their study had to keep the desired response in mind over a delay-period, and memory load is greater for 4 compared with 2 syllables.

In summary, we are arguing that enhanced pvSMG activation for sublexical phonological cues in the visual modality reflects auditory short-term memory. Other studies have shown that pvSMG activation is also enhanced during auditory short-term memory tasks on nonverbal stimuli (Koelsch et al. 2009). It is therefore not specific to speech sounds. Indeed, we found pvSMG activation to be highest during nonverbal auditory object naming (see Fig. 3).

## Anterior dorsal SMG

An anterior part of dSMG was more activated for reading pseudowords and naming objects than all other speech production conditions. The location of this pseudoword and object effect [at MNI −51, −30, 39] corresponds very closely to that reported in previous studies of phonological relative to semantic decisions on visual words [at MNI −52, −35, 40] as well as some of the studies comparing pseudoword to word reading [at MNI −49, −35, 39] (see Table 1). It also extended posteriorly and medially [at MNI −39, −33, 42] into the area associated with executive processing [at MNI −42, −37, 38 in Ravizza et al. 2004] and phonological decisionson words [−55, −35, 40] when semantic or executive processing is not controlled (Seghier et al. 2004).

Enhanced adSMG activation for pseudoword reading and object naming compared with word reading cannot be explained in terms of orthographic-to-phonological recoding because object naming involves no orthographic input but word reading does. We can also exclude explanations in terms of (1) phonological output which was matched in the reading and object naming conditions; (2) phonological short-term memory because adSMG activation was not higher for repetition and OB matching of auditory pseudowords than auditory object naming; and (3) visual attention because activation was not higher for visually presented pseudowords and objects than OB matching of the auditory baseline.

The observation that adSMG activation was as high for OB matching of the baseline conditions (color and gender) as it was for pseudoword reading may provide some clues to its function. Unexpectedly, the behavioral data (see results section for details) indicate that, during OB matching, accuracy is lower and RTs are highest for the baseline conditions, which involved matching 2 consecutive stimuli on the basis of perceptual features (color or gender). The longer RTs/loss of accuracy may have arisen because the same features were repeated multiple times in each scanning session (not just when a OB response was required) and this might have increased the level of interference or uncertainty relative to other conditions that did not involve multiple presentations of the same feature. Likewise, enhanced activation for pseudoword reading and object naming compared with word reading may reflect ambiguous, and thus more difficult, mappings between (1) sublexical orthography and phonology in the case of pseudoword reading, and (2) semantics and phonological outputs in the case of object naming (i.e., the same semantic concept can have multiple names). In contrast, word reading may be less ambiguous because it is constrained by both sublexical phonological cues and semantics.

Whatever its true function, the activation profile of the adSMG region across tasks cannot be explained in terms of phonological processing per se. Instead, we are proposing that previously reported adSMG activation for phonological compared with semantic decisions or pseudoword reading compared with word reading might reflect functions that are not specific to phonological processing but appear to be called on when there is ambiguity in the mapping between inputs (auditory and visual) and outputs.

Future studies could examine the function of adSMG more precisely by manipulating the ambiguity of sensory to motor mapping within task. This might explain why increased adSMG activation for pseudoword relative to word reading has not consistently been reported (see Table 1). It would also be informative to use functional connectivity studies (e.g., dynamic causal modelling (DCM)) to investigate how activity in adSMG links sensory inputs to motor outputs. Specifically, it would be useful to know whether adSMG is primarily driven top-down from motor and/or frontal regions and/or bottom-up from sensory input regions. For the time being, the current study contributes to our understanding by showing how adSMG activation varies across a range of different tasks; and how this pattern of response is functionally distinct from that of other SMG regions that also respond during word and pseudoword processing.

## Anterior ventral SMG

AvSMG showed 3 effects that were consistent with its role in phonological output processing irrespective of the presence or absence of phonological cues: It was (1) more activated for speech production than OB matching, (2) speech production activation was least for the baseline conditions (i.e., naming colors and gender) that involved repeatedly saying the same spoken response in the same scanning run and (3) activation was the same for conditions that were matched for articulatory output (i.e., word and object naming). Notably, avSMG activation did not differ significantly across object naming, reading, and repetition of familiar words and unfamiliar pseudowords. This allowed us to exclude a role for this area in (1) auditory short-term memory because activation related to auditory memory should be greater during auditory object naming than visual object naming; (2) orthographic to phonological mapping which would result in more activation for words than objects, (3) processing semantics which would result in more activation for objects than words or (4) managing task difficulty which would result in more activation for objects and pseudowords than words because behavioral evidence indicates that words are faster to process.

The avSMG that we associate with phonological output processing (at MNI coordinates [−57, −30, 27]) is ventral to the more dorsal anterior SMG activations that have previously been reported for phonological relative to semantic decisions, or reading pseudowords > reading familiar words (see Table 1). However, it is interesting to note that the avSMG region that we associated with phonological output processing corresponds more closely with that associated with phonological versus semantic decisions in transcranial magnetic stimulation (TMS) studies (e.g., Romero et al. 2006 with mean coordinates at [−46, −30, 26]; Sliwinska et al. 2012 at [−52, −37, 32]). Sliwinska et al. (2015) suggest that the stimulation over avSMG [−52, −34, 30] disrupted covert articulation. In which case, the claim would be that avSMG is more important (or necessary) for phonological than semantic decisions. The absence of significant avSMG activation in the comparison of phonological and semantic decisions in fMRI studies can also be explained if covert articulation occurred during both phonological and semantic decisions even though it was only necessary for phonological decisions.

## Posterior dorsal SMG

A lateral part of the pdSMG was more activated for reading and repeating words than all other speech production conditions. This is consistent with a role for this region in integrating lexical and sublexical phonological cues. An explanation in terms of semantic processing can be excluded because this should result in more activation for object naming that relies on semantic mediation than word repetition and reading that is facilitated by sublexical phonological information. To contrary, we found that pdSMG activation was less for object naming than repetition and reading. Instead, we found that increased demands on semantic processing (during object naming and word production) increased activation in the angular gyrus as reported previously (e.g., Price et al. 1997; Binder et al. 2003; Devlin et al. 2003; Diaz and McCarthy 2007; Seghier et al. 2010; Sharp et al. 2010). Thus, the pdSMG area that we are associating with the integration of lexical and sublexical inputs lies conveniently close but anterior to regions in the angular gyrus that are associated with semantic processing.

Anatomically, pdSMG has been shown to have direct cortico-cortical connections linking anteriorly to SMG and posteriorly to the angular gyrus (Lee et al. 2007). Cyto-architectonically, posterior SMG shows characteristics of both anterior SMG and anterior ANG and has therefore been described as a “transition zone” between these areas (Caspers et al. 2006). However, very little is known about the function of lateral pdSMG during word processing because it is rarely reported in functional imaging studies of language (Richardson et al. 2010). What we have previously reported is that gray matter in this region is higher in teenagers who have richer vocabularies (Lee et al. 2007; Richardson et al. 2010) and in adults who speak more than one language (Mechelli et al. 2005; Grogan et al. 2012). In Richardson et al. (2010), we suggested that pdSMG was involved in explicit vocabulary learning but this does not explain why we now report activation during word reading and repetition that do not involve such learning.

Clues to the function of lateral pdSMG come from the observation that it was as responsive during word repetition as it was during word reading. We suggest that it may be involved in the active process of integrating lexical and sublexical information during word repetition and reading; however, we do not know what type of lexical and sublexical information is being integrated (e.g., articulatory sequences or auditory associations). We think it is unlikely that lateral pdSMG activation reflects conflict between lexical and sublexical inputs because there is no prior evidence to suggest that activation in this area increases with the known conflict between lexical and sublexical cues during irregular word reading (e.g., Binder et al. 2005; Mechelli et al. 2005; Nosarti et al. 2010). Further studies of how pdSMG activation influences, and is influenced by, activation in other regions may provide more clarity on how it contributes to word processing.

## Conclusions

Our results have implications for differentiating different types of phonological input and output processing and the functional contributions of different SMG regions. As reported previously, we found that a posterior ventral part of SMG (on the border with the temporal lobe) is activated by tasks that increase demands on auditory short-term memory for verbal and nonverbal stimuli. In addition, we dissociate for the first time the following effects in different parts of SMG: (1) the ventral SMG region associated with articulatory output is anterior to that involved in auditory short-term memory; (2) a lateral part of pdSMG is involved in the integration of lexical and sublexical inputs and (3) activation in the adSMG that has previously been associated with phonological relative to semantic decisions and for reading pseudowords compared with words, could not be explained in terms of phonological processing but appeared to be involved in more difficult tasks, i.e., when there was ambiguity in the mapping between sensory inputs and motor outputs.

Effective connectivity studies, using techniques such as DCM, could take our findings a step further and explore the connections of different parts of SMG with other cortical areas, and their precise roles within the distributed network of phonological processing. Our findings could also be tested by comparing the consequences of focal TMS or permanent brain damage to each of the SMG sub-regions during a range of different tasks. For example, does selective disruption to posterior SMG differentially impair word repetition and reading?

## Funding

Wellcome Trust (097720).
